# Analysis of glutathione and vitamin C effects

**DOI:** 10.1100/tsw.2006.205

**Published:** 2006-09-25

**Authors:** M. Pitarque, A. Creus, R. Marcos

**Affiliations:** Grup de Mutagènesi, Departament de Genètica i de Microbiologia, Edifici Cn, Universitat Autònoma de Barcelona, 08193 Bellaterra, Spain

**Keywords:** comet assay, oxidative DNA damage, benzenetriol, glutathione, vitamin C

## Abstract

The alkaline single-cell gel electrophoresis (or Comet) assay was applied to evaluate the eventual DNA damage induced by the triphenolic metabolite of benzene, 1,2,4-benzenetriol (BT), in isolated human lymphocytes. Prior to BT treatment, ranging from 5 to 50 μM, a supplementation with glutathione (GSH, 350 μg/ml) was carried out to assess whether GSH may have a modulating effect on the Comet response. The effect of a fixed dose of BT was also evaluated in the presence of the exogenous antioxidant vitamin C (40 and 200 μM). Additionally, we investigated whether the polymorphism of glutathione S-transferase T1 (*GSTT1*) gene may affect the individual level of BT-induced DNA damage *in vitro*. For all donors included in the present study, BT produced a significant dose-response relationship. No clear effect of GSH preincubation was seen on the BT-induced response. On the contrary, a significant reduction of DNA damage was observed in the presence of vitamin C (at least at 200 μM). Although our data suggest some individual differences according to the *GSTT1* genotype in the outcome of the Comet assay, a large number of individuals should be studied in further investigations to obtain reliable conclusions.

## INTRODUCTION

Benzene is a well-known promutagen and benzene metabolites are responsible not only for its genotoxicity, but also for its myelotoxicity and leukemogenesis[1,2]. The majority of benzene metabolism occurs in the liver, where cytochrome P4502E1 catalyzes the first step leading to the formation of phenol and other subsequent metabolites as cathecol, hydroquinone, and benzenetriol (BT). In addition, it has also been demonstrated that secondary metabolism of benzene metabolites in bone marrow critically contributes to benzene toxicity. The phenolic metabolites and muconic acid accumulate in the bone marrow, where myeloperoxidases may further convert them to their corresponding quinone and semiquinone radicals[3,4,5]. These can covalently bind to DNA and other cellular macromolecules, and are generally believed to be the ultimate toxic and genotoxic species.

BT, a minor and highly unstable triphenolic metabolite of benzene, has a strong ability to bind transition metal ions and rapidly autoxidizes to 2-hydroxy-1,4-benzoquinone and its semiquinone radical intermediates. Moreover, during this process, the products of BT oxidation have the potential to undergo cyclic reduction and oxidation reactions (redox cycling) that generate large amounts of reactive oxygen species, including superoxide anion ($O_2^-$), hydrogen peroxide ($H_2O_2$), and hydroxyl radical ($OH^\bullet$), as well as perhaps singlet oxygen ($^\bullet O_2$). These reactive species are known to damage DNA and other cellular macromolecules[6,7,8]. Thus, oxidative mechanisms leading to a broad spectrum of DNA damage[9,10,11], as well as quinones-related microtubule disruption[12,13,14], have been reported as different pathways for the induction of genetic damage by BT.

DNA damage due to the attack of free radicals or the action of electrophiles may be affected by GSH, which may play an important role in antimutagenesis and anticarcinogenesis as a free radical scavenger[15]. GSH along with the multigene family of phase II detoxifying enzymes, glutathione S-transferases (GST), are assumed to constitute one of the more important systems that prevent mutagenic elctrophiles from being carcinogenic[16].

GSTs catalyze reactions of GSH with different exogenous electrophilic substrates and the GSH conjugates formed are usually nontoxic excretory products. By means of their GSH peroxidase activity, these enzymes also detoxify endogenous lipid and DNA hydroperoxides arising from oxidative damage[16,17,18]. It is known that some polyaromatic hydrocarbons, benzene metabolites, and their oxidation products may covalently bind to GSH and form nontoxic GSH conjugates[1,19]. Thus, genetic polymorphisms of GST and other xenobiotic-metabolizing enzymes that may influence the individual ability to activate or detoxify different benzene metabolites, including BT and its highly reactive quinone and semiquinone radicals, might possibly affect the amount of genetic damage formed and thereby modulate the susceptibility to benzene-induced mielotoxicity and leukemia[20].

Vitamin C can modify responses to oxygen radicals, acting under different conditions as either a radical scavenger or a pro-oxidant, producing hydrogen peroxide and free radicals by autoxidation[21,22]. A protective effect against oxidative damage and benzo(α)pyrene was observed in human lymphocytes both after *in vitro* and *in vivo* treatments[23,24,25].

The alkaline single-cell gel electrophoresis (or comet) assay (SCGE or CA) has been demonstrated to be a very sensitive method for measuring DNA single strand breaks and alkali-labile sites at the individual cell level[26,27]. This method previously revealed significant responses in human lymphocytes after *in vitro* treatments with BT and other benzene metabolites[28,29]. Using the same type of cells, the CA was applied to assess the modulating or protective effect of both GSH and vitamin C on BT genotoxicity, taking also into account the possible influence of *GSTT1* genotype.

## MATERIALS AND METHODS

### Blood Donors, Lymphocyte Isolation, and Genotype Analyses

Seven young men were used in the present study as blood donors. All of them were nonsmoking healthy people and ranged in age from 19–27 years. Prior to the CA experiments, genomic DNA extracted from leukocytes by standard methods was used as a template in the *GSTT1* genotype analyses described elsewere[30]. Heparinized venous blood samples were drawn from each donor and lymphocytes were isolated immediately using a Ficoll density gradient. Approximately 15 ml of whole blood was diluted (1:1) with phosphate buffered saline (PBS) and centrifuged over 15 ml of lymphocyte isolation medium (Rafer, Zaragoza, Spain) at 800 *g* for 30 min. Buffy coat was removed and washed twice with PBS at 400 *g* for 10 min, then the cells were resuspended in RPMI 1640 medium (Gibco BRL, Paisley, Scotland, U.K.) and counted in a hemocytometer.

## Treatments

Isolated lymphocytes ($10^6$ cells/ml) were dispensed to Eppendorf tubes in RPMI 1640 medium without serum. In experiment 1, lymphocyte samples were preincubated at 37ºC for 30 min, with and without GSH (350 μg/ml). After this, appropriate volumes of BT were added to attain 5, 10, 25, and 50 μ*M* concentrations. In experiment 2, lymphocytes were first incubated with vitamin C (0, 40, or 200 μ*M*) at 37ºC for 30 min, and a subsequent treatment with BT (25 μ*M*) was carried out. In both experiments, treatments were done for 30 min at 37ºC and $H_2O_2$ (20 μ*M*) was used as positive control. After treatment, cells were washed with PBS, centrifuged, and the pellet was resuspended in PBS.

## Single-Cell Gel Electrophoresis

The CA was performed under alkaline conditions, basically as described by Singh et al.[31]. In brief, 80 μl of 0.7% low-melting-point agarose (LMA in $Ca^{2+}$-$Mg^{2+}$-free PBS at pH 7.4, 37ºC), containing about $2 \times 10^5$ cells, were pipetted onto a roughened slide precoated with a 125-μl layer of 1% normal-melting-point agarose (NMA). The agarose was allowed to set on ice for 5 min. A third layer of 80 μl of LMA was applied and solidified again on ice. After removal of the coverslip, the slides were immersed in freshly prepared lysing solution (2.5 *M* NaCl, 100 m*M* $Na_2$EDTA, 10 m*M* Tris-HCl, pH 10, with 1% Triton X-100 added just before use) for a minimum of 1 h at 4ºC after which they were placed in a horizontal electrophoresis tank in an ice bath. The tank was filled with freshly made alkaline buffer (1 m*M* $Na_2$EDTA, 300 m*M* NaOH, pH 13), and the slides were left in the high-pH buffer for 20 min to allow DNA unwinding and expression of alkali-labile sites. Electrophoresis was carried out for 20 min at 25 V and 300 mA in the dark. The slides were then neutralized by adding dropwise 0.4 *M* Tris-HCl (pH 7.5, washed three times for 5 min each).

We purchased BT and GSH from Aldrich Chemical Co. Ltd. (Gillingham-Dorset, U.K.), and PBS tablets and vitamin C from Sigma Chemical Co. (St. Louis, MO). Fetal calf serum, NMA, and LMA were obtained from Gibco BRL.

## Staining and Slide Scoring

The slides were stained with 60 μl of a 4’,6-diamidino-2-phenylindole dihydrochloride (DAPI) solution at a concentration of 5 and 0.2 μg/ml, for experiments 1 and 2, respectively. The slides were kept in a humidified chamber until scoring.

In experiment 1, the slides were examined by eye using an Olympus BX50 fluorescence microscope equipped with a wide-band excitation filter of 330–385 nm and a barrier filter of 420 nm. One hundred cells (50 per replicate) were scored at 400× magnification, and the mean comet length was calculated for each concentration. Moreover, cells were graded by eye into five categories (A–E) according to the amount of DNA in the tail[23], where A are undamaged cells and E highly damaged cells. From the arbitrary values assigned to the different categories (from A = 0 to E = 4), a genetic damage index (GDI) was calculated.

In experiment 2, the slides were examined using a Komet 3.1 image analysis system (Kinetic Imaging Ltd., Liverpool, U.K.). One hundred cells (50 per replicate) were scored for each concentration, and the mean tail moment (TM) and the percentage of DNA in the tail (%TDNA) were measured as DNA damage parameters.

## Statistical Analysis

In all cases, significant correlation coefficients ($r > 0.900$; $p < 0.001$) were found between comet length and GDI, and also between TM and %TDNA (data not shown). For all donors included in this study, the significance of the dose-response relationship was evaluated by means of a regression analysis, and CA values obtained from each BT treatment were compared with its negative control by means of the nonparametric Mann-Whitney U test. The same test was used to assess the possible modulating effect of both GSH and vitamin C, comparing DNA damage results between preincubated lymphocytes and their parallel cultures with neither GSH nor vitamin C supplementation. *P* values lower than 0.05 were regarded as statistically significant. The results were analyzed using the CSS: STATISTICA/WTM (StatSoft, Tulsa, OK) statistical package.

## RESULTS

The characteristics of the donors included in the present investigation are summarized in Table 1. These individuals were selected from a larger group of potential donors according to health status, smoking habits, and *GSTT1* genotype.

**TABLE 1**
**Blood Donors Included in the Present Study**

| Donor | Age | *GSTT1* | Experiment 1, BT + GSH | Experiment 2, BT + Vit C |
|---|---|---|---|---|
| A | 19 | + | Yes | Yes |
| B | 20 | + | Yes | – |
| C | 27 | + | Yes | Yes |
| D | 20 | Null | Yes | Yes |
| E | 19 | Null | Yes | Yes |
| F | 20 | Null | Yes | – |
| G | 26 | + | – | Yes |

As can be seen in Table 2, a statistically significant dose-response relationship was observed for the six donors included in the experiment 1, and Fig. 1 shows the mean comet length values obtained from each treatment. Both in the absence and presence of GSH, BT significantly induced DNA damage. In some donors, this increase could be detected even at the lowest doses tested (5 and 10 μ*M*). An important variability for the magnitude of the test outcome was observed between the different donors, some of them (B and C) being more affected by BT treatments than others (D and E).

Although a slight trend towards the reduction of BT-induced DNA damage was observed when preincubating lymphocytes with GSH (mainly in donors C, D, and E), the possible modulating effect of GSH was not clearly revealed using the CA. When tested alone at a concentration of 350 μg/ml, GSH failed to induce DNA damage.

In the same experiment, when cells were pooled according to the *GSTT1* genotype, *GSTT1* + donors apparently showed to be more sensitive than *GSTT1* null donors to *in vitro* BT effects, both in the absence and presence of GSH (Fig. 2).

The response to a fixed concentration of BT (25 μ*M*) or $H_2O_2$ (20 μ*M*), in combination with different concentrations of vitamin C, was evaluated using the CA in experiment 2. The mean TM values for all donors are showed in Fig. 3. The clear BT and $H_2O_2$ positive results obtained in this second experiment confirmed those previously obtained when scoring by eye. Between the different donors, there were some differences in the degree of DNA damage induced by BT and $H_2O_2$, being the lymphocytes from individuals C and G those most affected by treatments.

**TABLE 2**
**Regression Coefficients (β) Between Comet Length Values and BT Concentrations**

| Donor | Without GSH | With GSH |
|---|---|---|
| A | 0.661 | 0.751 |
| B | 0.549 | 0.713 |
| C | 0.692 | 0.653 |
| D | 0.703 | 0.658 |
| E | 0.656 | 0.587 |
| F | 0.668 | 0.698 |

Coefficients are statistically significant ($p < 0.001$).

At least in three donors (A, E, and G), vitamin C (200 μ*M*) alone produced statistically significant increases when compared with negative controls. Only donor C showed a significant decrease in basal DNA damage after preincubation with vitamin C. As can be seen in Fig. 3, lymphocytes from all donors (except donor A) showed a significant vitamin C–related reduction in the level of oxidative damage induced by BT and $H_2O_2$. This protective effect was seen mainly at 200 μ*M*, but even at 40 μ*M* in some cases.

## DISCUSSION

Measuring increased 8-hydroxy-2'-deoxyguanosine levels, BT was found to be the most effective phenolic benzene metabolite inducing oxidative DNA damage when administered alone both in HL-60 cells *in vitro* and in mouse bone marrow *in vivo*[13,32]. It has been suggested that the ability of BT to induce apoptosis in human bone marrow progenitor cells may also be involved in benzene myelotoxicity[33]. It is known that autoxidation and peroxidative metabolism of the phenolic metabolites of benzene produce active oxygen[5], which is capable of causing oxidative DNA lesions and breaks[6,7]. In the case of BT, the autoxidation process readily occurs due to its instability and it is enhanced by metal ions[9,10,11,13,34]. In a series of studies using HL-60 cells, BT increased superoxide and $H_2O_2$ production, and decreased superoxide dismutase and sulhydryl levels, but not catalase activity[35].

With regard to the DNA-binding capacity of quinone and semiquinone radicals produced during BT metabolism as one of the proposed mechanisms for BT genotoxicity[1,13], the time dependency of DNA adduct formation by BT in HL-60 cells is consistent with the process of BT myeloperoxidative activation[36]. In the same study, the authors suggested that the synergistic interaction of benzene metabolites in the production of DNA adducts may have significant biological effects. Moreover, for the same level of cytotoxicity, BT was found to be a less-effective inducer of DNA adducts than hydroquinone, indicating that cellular mechanisms other than adduct formation contribute to the cytotoxicity of BT. Similarly, Hedli et al. indicated that the lack of BT-related DNA modification in HL-60 cells would suggest that DNA adducts not play a role in inhibiting cell differentiation in BT-treated cells[37]. According to Lee and coworkers, BT and other benzene metabolites might be able to induce DNA strand breaks in living cells without involving DNA adduct formation, by means of the reactive oxygen species generated which are also responsible for the inhibitory effect of BT on DNA synthesis, in addition to their inhibitory effect on DNA polimerase α[38,39].

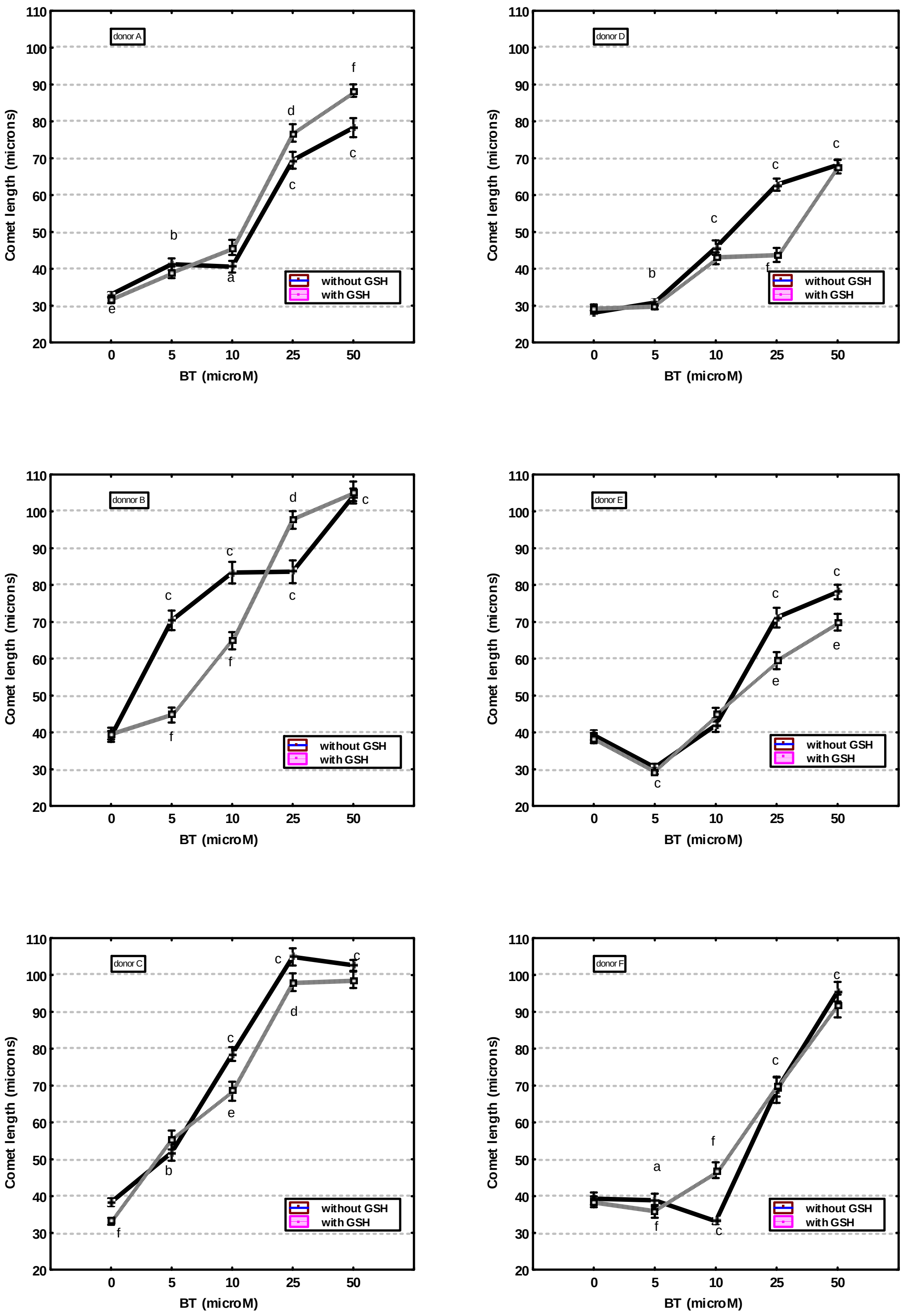

**FIGURE 1.** Mean comet length values for the six donors included in the experiment 1. BT treatments in absence of GSH were compared with control values by means of the Mann-Whitney U test ($^{a}p < 0.05$, $^{b}p < 0.01$, $^{c}p < 0.001$). Using the same test, DNA damage values in GSH preincubated lymphocytes were compared with values in their parallel nonsupplemented cells ($^{d}p < 0.05$, $^{e}p < 0.01$, $^{f}p < 0.001$).

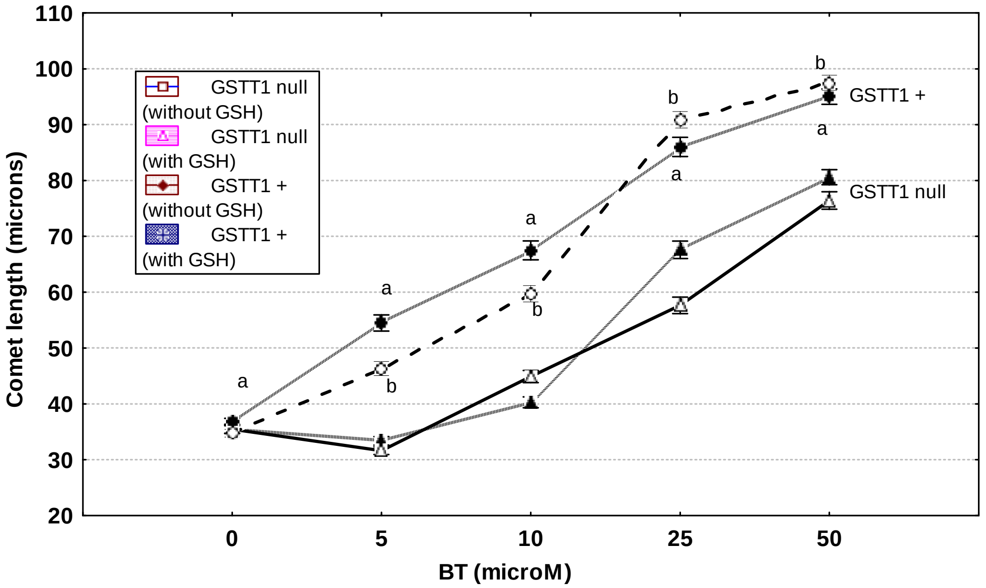

**FIGURE 2.** Mean comet length values in *GSTT1* + (A, B, and C) donors compared with those in *GSTT1* null (D, E, and F) donors. Differences between genotypes are statistically significant ($p < 0.001$; Mann-Whitney U test) both in the absence ([a]) and presence ([b]) of GSH.

In general, BT appears to consistently test positive as a genotoxic agent and it is believed to play some role in the genotoxicity observed following benzene exposure. Some studies have shown that BT induces alkali-labile sites and DNA breakage, mostly through the generation of oxygen radicals[9,40]. BT also induced significant increases in the SCE frequency in human lymphocytes[41] and V79 cells, as well as mutations to 6-thioguanine resistance gene[42]. Using an antikinetochore antibody-based micronucleus assay and FISH techniques in HL-60 cells, BT was found to produce mainly aneugenic effects by disrupting microtubules[13,14]. Moreover, *in vitro* inhibition of enzymes involved in DNA replication and repair, as topoisomerase enzymes, by BT and other benzene metabolites represents a potential mechanism for the formation of chromosomal aberrations and may contribute to the leukemogenic effect of benzene[43,44].

Considering all these data on the cytotoxic and genotoxic effects of BT, and taking into account the ability of the alkaline SCGE in detecting oxygen-radical-generated DNA damage, our positive CA results were certainly expected and confirmed that BT is able to induce dose-dependent responses in human lymphocytes. The DNA-damaging ability of BT was potent enough to be detected in some cases at a concentration as low as 5 μ*M*. Specifically, our results are in agreement with those obtained in *in vitro* CA experiments by Anderson et al.[28] and Andreoli et al.[29].

Glutathione is one of the most important compounds for maintaining the cellular integrity due to its reducing property, which allows it to modulate the redox status of metal ions and other agents and hence affect the mode and the rate of reactive oxygen species generation[45]. According to our results, preincubating human lymphocytes during 30 min with GSH (350 μg/ml) did not modify the level of BT-induced DNA damage, indicating that, in this *in vitro* protocol, GSH does not act as a reductor agent. One feasible explanation for this apparent failure of GSH as a protective agent could be that in the presence of oxygen, at least for some cell types, GSH is no longer protective, as it has been found in some radiosensitivity studies using GSH-deficient cells or GSH-depleted cells[46,47]. Our results are not in accordance with previous observations in human bone marrow cells, showing that a similar 30-min preincubation with GSH (350 μg/ml, the average human blood concentration) completely blocked the DNA damaging effect of benzoquinone and reached a 53% blockage in the case of BT[41]. However, the opposite effect was observed in a chemical system, where GSH (1 m*M*) enhanced the extent of 8-DNA double strand breaks induced by hydroquinone, BT and vitamin C[48].

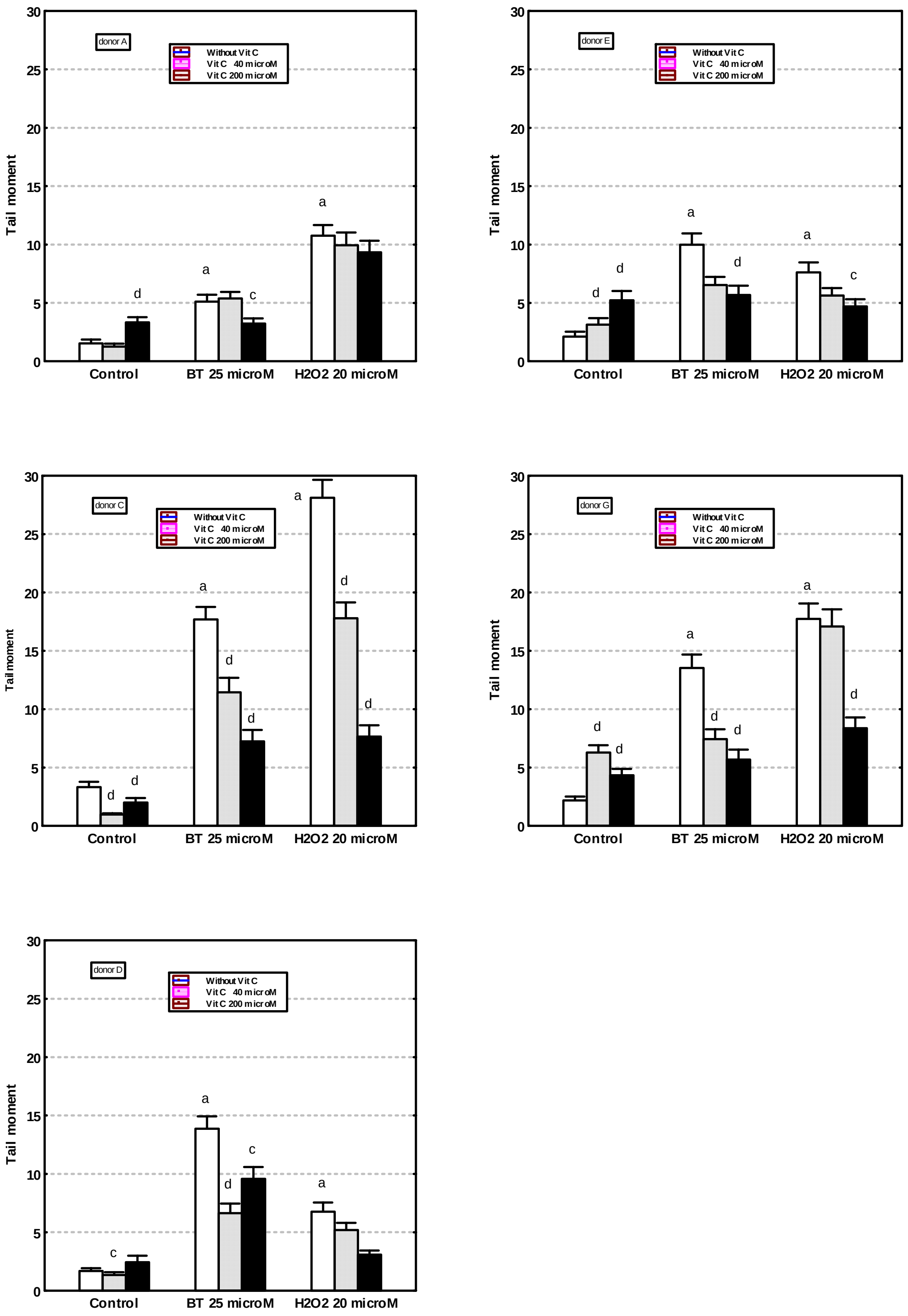

**FIGURE 3.** Mean tail moment values from combined treatments with BT (25 μ*M*) or $H_2O_2$ (20 μ*M*), and vitamin C supplementation (0, 40, and 200 μ*M*) for the five donors. BT or $H_2O_2$ alone significantly increased DNA damage levels ($^{a}p < 0.001$; Mann-Whitney U test). The same test was also used to assess the antioxidant effect of vitamin C ($^{b}p < 0.01$, $^{c}p < 0.001$).

In our study, when it was tested alone, vitamin C (200 μ*M*) induced slight, but significant, increases in DNA damage, probably acting as an electron donor and accelerating reactive oxygen species generation *in vitro* under aerobic conditions. On the other hand, at the same concentration, acting as a free radical scavenger, vitamin C decreased the oxidative DNA damage levels when it was added 30 min before BT or $H_2O_2$. For the five donors included in the second experiment, the potency of the protective effect at 200 μ*M* was calculated as a percentage of damage from unopposed treatments, ranging from 40–69% and from 27–87% for BT and $H_2O_2$, respectively. This *in vitro* antioxidant effect on $H_2O_2$-induced DNA damage was also observed by Noroozi et al.[49]. However, using a similar protocol, vitamin C in combination with $H_2O_2$ had a small protective effect at low doses and exacerbated effects at high doses[23]. Applying an *ex vivo* method, lymphocytes of vitamin C–supplemented subjects showed an increase resistance to baseline and induced oxidative damage[50,51]. In addition, vitamin C also affect DNA repair[52,53].

In the present investigation, responses to BT treatments were somehow consistently reproduced in six different donors, although certain differences could be detected when their *GSTT1* genotypes were considered. In fact, lymphocytes from *GSTT1* + subjects seemed to be more sensitive to BT action than those from *GSTT1* null subjects (i.e., individuals lacking GSTT1 enzyme). This result would be strange or at least unexpected because, theoretically, individuals who lack a specific GST enzyme are particularly vulnerable to adverse health effects if exposed to its harmful substrates[16,20,54] and GSTs are supposed to interact with reactive metabolites in the latest steps of benzene metabolism[1]. It is not clear yet whether covalent binding and activation by BT have implications in the regulation of the microsomal GST and, since some phenol reactive metabolites might be substrates for the enzyme, their elimination through conjugation[55]. Anyway, we recognize that the fact that we have used isolated lymphocytes could mislead us to the possible effect of *GSTT1* genotype, since the activity of this enzyme is expressed mainly in erythrocytes[20,56]. In short, because of the low number of donors, we are not allowed to conclude firmly that *GSTT1* polymorphism may lead to individual susceptibility differences in the genotoxic response to BT, but this observation deserves further investigation and other GST genotypes should be also analyzed.

## ACKNOWLEDGMENTS

This investigation has been supported in part by a grant from the Generalitat de Catalunya (CIRIT, 2005SGR-00136). We would like to thank G. Umbert for their expert technical assistance.
